# Predictive nomogram for postoperative pancreatic fistula following pancreaticoduodenectomy: a retrospective study

**DOI:** 10.1186/s12885-021-08201-z

**Published:** 2021-05-15

**Authors:** Jian Shen, Feng Guo, Yan Sun, Jingyuan Zhao, Jin Hu, Zunxiang Ke, Yushun Zhang, Xin Jin, Heshui Wu

**Affiliations:** 1 Department of Pancreatic Surgery, Union Hospital, Tongji Medical College, Huazhong University of Science and Technology, Wuhan, 430022 China; 2 Department of Breast and Thyroid Surgery, Union Hospital, Tongji Medical College, Huazhong University of Science and Technology, Wuhan, 430022 China; 3 Department of Vascular Surgery, Union Hospital, Tongji Medical College, Huazhong University of Science and Technology, Wuhan, 430022 China; 4 Cancer Center, Union Hospital, Tongji Medical College, Huazhong University of Science and Technology, Wuhan, 430022 China

**Keywords:** Albumin, BMI, Drain amylase value, Pancreatic texture, Risk factors

## Abstract

**Background:**

Postoperative pancreatic fistula (POPF) represents the most common complication following pancreaticoduodenectomy (PD). Predictive models are needed to select patients with a high risk of POPF. This study was aimed to establish an effective predictive nomogram for POPF following PD.

**Methods:**

Consecutive patients who had undergone PD between January 2016 and May 2020 at a single institution were analysed retrospectively. A predictive nomogram was established based on a training cohort, and Lasso regression and multivariable logistic regression analysis were used to evaluate predictors. The predictive abilities of the predicting model were assessed for internal validation by the area under the receiver operating characteristic curve (AUC) and calibration plot using bootstrap resampling. The performance of the nomogram was compared with that of the currently used a-FRS model.

**Results:**

A total of 459 patients were divided into a training cohort (*n* = 302) and a validation cohort (*n* = 157). No significant difference was observed between the two groups with respect to clinicopathological characteristics. The POPF rate was 16.56%. The risk factors of POPF POPF were albumin difference, drain amylase value on postoperative day 1, pancreas texture, and BMI, which were all selected into a nomogram. Nomogram application revealed good discrimination (AUC = 0.87, 95% CI: 0.81–0.94, *P* <  0.001) as well as calibration abilities in the validation cohort. The predictive value of the nomogram was better than that of the a-FRS model (AUC: 0.87 vs 0.62, *P* <  0.001).

**Conclusions:**

This predictive nomogram could be used to evaluate the individual risk of POPF in patients following PD, and albumin difference is a new, accessible predictor of POPF after PD.

**Trial registration:**

This study was registered in the Chinese Clinical Trial Register (ChiCTR2000034435).

## Background

Pancreaticoduodenectomy (PD) is the only potentially curative procedure for the treatment of tumours located in the pancreatic head and periampullary region [1, 2]. According to reports, the incidence rate of pancreatic cancer [3] and tumours of the periampullary area [4] are increasing year by year, which also means that cases of pancreaticoduodenectomy are increasing [5]. However, the incidence of postoperative complications after PD is still up to 70% [2, 5]. Postoperative pancreatic fistula (POPF) is one of the most severe and frequent complications following PD, the rate of which is up to 30% [6–8]. The leakage of pancreatic juice destroys the surrounding tissues and is a main causal factor of several ensuing complications, such as postoperative abdominal bleeding, abdominal abscess, organ dysfunction, and even death [1, 2, 7–10].

Many measures have been taken to reduce the occurrence of POPF, among which surgical techniques [11], the usage of additional materials [12], and somatostatin and its analogs [13] have attracted much attention. However, data regarding the optimal management for the prevention of POPF have still not been established [11, 12]. The accurate selection of patients with a high risk of POPF at an early stage is important for optimizing the perioperative management of these patients [6, 9].

Numerous risk factors for POPF have been identified, such as sex, pancreatic texture, blood loss, and main pancreatic duct diameter; however, most of these predictors are subjective and contradictory [10]. Several predictive models have also been proposed, among which the fistula risk score and alternative fistula risk score (a-FRS) are most commonly used [9, 10]. However, the performances of those models were recently suggested to have low predictive value for external validation [9, 10, 14, 15]^.^ New objective predictors are needed to build a reliable model [10, 13]. Many biochemical parameters are routinely tested in the clinic, such as albumin, the level of which declines after surgery. Studies have demonstrated that the difference between the levels of preoperative and postoperative albumin is associated with postoperative complications [16–18], but little is known about its value for predicting POPF following PD. This study aimed to establish an effective predictive nomogram, including the albumin difference, for POPF following PD to help doctors screen high-risk patients on postoperative day (POD) 1.

## Methods

### Patient cohort and study design

A retrospective study of all consecutive patients undergoing PD was analysed from January 2016 to May 2020. Patients who met the following criteria were excluded: those with a history of pancreatectomy; combined with distal pancreatectomy; history of chronic organ insufficiency; less than 18 years old; with total pancreatectomy or duodenum-preserving pancreatic head resection.

The patients were divided into two cohorts: the training cohort contained patients from 2016 to 2018, and the validation cohort included patients from January 2019 to May 2020. Written informed consent forms were signed before the operation. The work has been reported in line with the STROCSS criteria [19] and obeys the TRIPOD statement.

### Operative intervention

Physiological and psychological adjustments were made for all the patients after admission. The surgical methods of an open approach and minimally invasive surgery were included in this study. The surgical approaches were performed by experienced pancreatic surgery surgeons and followed specific principles and guidelines, but particular techniques, such as pancreatic transection, were chosen according to the intraoperative situation. In patients with malignant neoplasms, intraoperative frozen sections were obtained to ensure a negative margin status, and regional lymphadenectomy was performed. A polyethylene stent tube was inserted into the pancreatic duct. The Child’s type digestive tract reconstruction technique was applied in all patients, and an end-to-side duct-to-mucosa pancreaticojejunostomy was performed. The pancreatic texture was divided into soft and hard groups based on intraoperative palpation by more than two experienced surgeons. Except for artificial blood vessels, no additional biological materials were used. The nasogastric tube (NGT) was placed during the operation. Routine peritoneal drainage tubes were placed adjacent to the pancreatoenteric anastomosis and the bilioenteric anastomosis.

### Postoperative care

Drugs inhibiting gastric acid secretion were routinely administered, while somatostatin and its analogs were not. Laboratory results, including routine blood and biochemical examinations, were collected on POD 1. All patients in the study underwent abdominal computed tomography (CT) or trans-abdominal ultrasound during the first week after surgery. Additional abdominal imaging was performed when there was a sign of possible intra-abdominal complications. The NGT was removed when the patients had positive intestinal movement or a drainage volume less than 500 ml per day. Oral diet, walking, and early withdrawal of drainage tubes were advocated at an early stage. Patients in good clinical condition but with biochemical fistula were discharged home with drainage tubes, which were removed during their follow-up when the fistula had disappeared. The patients were followed via outpatient, telephone, or network visits for at least 90 days.

### Data collection and definitions

The primary outcome was POPF. Pre-, intra- and postoperative data were collected based on previous suggestions or data thought to be clinically important. The diameter of the main pancreatic duct at the line where the pancreas was to be transected was measured on preoperative CT images.

POPF was defined based on the criteria of the 2016 International Study Group of Pancreatic Surgery as any drain amylase value (DAV) more than 3 times the reasonable upper limit of the serum amylase level on or after POD 3 associated with a clinically relevant change in management [20]. The normal upper limit of serum amylase is 135 U/L in our institution. A DAV greater than 405 U/L was classified as POPF if one of the following criteria was met: persisting drainage for more than 21 days; changes in clinical management strategies due to POPF; requirement for percutaneous, endoscopic interventions or reoperation; infection related to POPF; or organ failure or death.

In this article, the albumin difference (AD) was defined as the level of preoperative albumin minus postoperative albumin on POD 1.

### Statistical analysis

Continuous parameters were presented as the means with standard deviations or medians with ranges as appropriate. Normally distributed data were compared with t-tests, while nonnormally distributed data were evaluated by the Mann-Whitney U test; categorical variables were expressed as frequencies or percentages and analysed with the chi-squared or Fisher’s exact test.

Lasso regression, a very popular feature selection technique, was used to narrow the scope of the candidate parameters considered to be clinically important [21, 22]. The selected predictors were entered into the multivariable logistic regression analysis, and the results were expressed as adjusted odds ratios (ORs) with 95% confidence intervals (CIs). Predictors with *P* < 0.05 were applied to establish a predictive nomogram for POPF following PD.

The area under the curve of the ROC (AUC) and calibration curves via 1000 bootstrap resampling was used to assess the predictive accuracy and discriminatory capability of the nomogram. The difference between the AUCs was compared by using the DeLong test. The missing data were handled by multiple imputations. Statistical analysis was performed using SPSS (version 22, IBM Corp, Armonk, NY) and R software (version 3.6.2) with the *rms, glmnet and Hmisc* packages. A *P-*value of < 0.05 was considered statistically significant.

## Results

Data of 501 consecutive cases were retrieved from the hospital record database. 42 cases were excluded according to the exclusion criteria as follows: 16 because of a history of pancreatectomy, 4 owing to the combination of distal pancreatectomy, 14 because of duodenum-preserving pancreatic head resection, 2 owing to total pancreatectomy, and 6 because they were < 18 years old. Finally, 459 patients were included in the study cohort. Of these 459 patients, 302 were assigned to the training cohort, whereas 157 were assigned to the validation cohort. Pancreatic texture and DAV were missing in 11.98 and 6.97% of the cases, respectively; the other missing data accounted for < 5% of the cases.

### Clinicopathological characteristics

For the 302 patients in the training cohort, the mean BMI was 22.39 ± 2.78, the mean age was 56.47 ± 11.17 years, and 59.93% of these patients were male. The most common comorbidity was hypertension (18.21%), which was followed by diabetes mellitus (14.24%). A total of 57 patients (18.87%) had a history of upper abdominal surgery, and 31 patients (10.26%) had undergone preoperative biliary drainage. The serum total bilirubin level was slightly elevated because of biliary tract obstruction, and the POPF rate was 16.56%. No significant difference in clinicopathological characteristics was found between the training and validation cohorts (*P* > 0.05). A detailed summary of the clinicopathologic clinicopathological of the patients is shown in Table 1.

**Table 1:** Clinicopathological characteristics of patients (*n* = 459)

Variables	Training Cohort			Validation Cohort (*n* = 157)	*P* (Training Cohort vs Validation Cohort)
	Total (*n =* 302)	POPF (*n* = 50)	No-POPF (*n* = 252)		
Age (years)	56.47 ± 11.17	56.74 ± 11.94	56.42 ± 11.03	57.42 ± 10.77	0.380
BMI (kg/m^2^)	22.39 ± 2.78	23.39 ± 2.78	22.21 ± 2.75	22.34 ± 3.02	0.873
Sex					0.763
Female	121	18	103	60	
Male	181	32	149	97	
Smoking					0.999
Yes	92	16	76	48	
No	210	34	176	109	
Drinking					0.128
Yes	76	12	64	29	
No	226	38	188	128	
Biliary drainage					0.403
Yes	31	3	28	12	
No	271	47	224	145	
Diabetes					0.183
Yes	43	3	40	15	
No	259	47	212	142	
Hypertension					0.362
Yes	55	8	47	23	
No	247	42	205	134	
Epigastric operation history					0.704
Yes	57	13	44	27	
No	245	37	208	130	
Total bilirubin (μmol/L)	28.80 (14.75–148.10)	35.00 (10.20–168.13)	26.70 (13.13–147.85)	17.80 (10.85–151.20)	0.204
Albumin (g/L)	39.16 ± 3.82	39.90 ± 3.98	39.02 ± 3.73	39.51 ± 4.41	0.369
White blood cell (10^12^/L)	5.77 ± 1.60	5.76 ± 1.48	5.77 ± 1.61	5.75 ± 1.83	0.927
Platelet count (10^9^/L)	231.12 ± 84.20	239.12 ± 99.49	229.53 ± 89.95	242.77 ± 77.58	0.149
Operative time (min)					0.768
> 300 min	144	27	117	72	
≤ 300 min	158	23	135	85	
Pancreas duct size					0.555
> 3 mm	160	31	111	79	
≤ 3 mm	142	19	141	18	
Surgical procedure					0.526
Minimally invasive	58	9	49	26	
Laparotomy	224	41	203	131	
Pancreas texture					0.768
Soft	141	33	108	71	
Hard	161	17	144	86	
Pathology					0.207
Malignant tumour	212	29	183	101	
Other	90	21	69	56	
Tumour location					0.605
Pancreatic head	222	35	187	126	
Duodenal papilla	35	6	29	13	
Distal bile duct	19	3	16	8	
Ampulla	19	5	14	8	
Other	7	1	6	2	
Data on POD 1					
White blood cell (10^12^/L)	12.97 ± 4.00	12.41 ± 4.43	13.08 ± 3.91	13.16 ± 4.23	0.636
Platelet count (10^9^/L)	180.03 ± 60.57	169.68 ± 58.15	182.08 ± 60.95	187.15 ± 64.74	0.244
AD (g/L)	10.81 ± 4.22	12.94 ± 4.65	10.39 ± 4.00	10.77 ± 4.13	0.913
Drain amylase value (U/L)	243.5 (54.00–1436.8)	932.5 (347.00.00–6091.00)	169.50 (43.25–984.75)	242.00 (49.00–1502.00)	0.907

### Parameter selection

A total of 23 candidate parameters (Table 1) in the training cohort were screened and verified using Lasso regression, and five-fold cross-validation through the minimum criteria was used. Finally, seven potential predictors, BMI, operative time, pancreatic texture, main pancreatic duct size, AD, DAV on POD 1, and pathology, were selected and incorporated into the logistic regression analysis. The results showed that AD, pancreatic texture, DAV, and BMI were independently correlated with POPF (Table 2).

**Table 2:** Multivariate analysis of predictors of POPF (*n* = 302)

Variables	β	OR	95% CI	*P*
Albumin difference (change per 5 points)	0.61	1.83	1.30–2.59	0.001
Drain amylase value (change per 5000 points)	0.29	1.33	1.06–1.68	0.015
BMI	0.11	1.15	1.03–1.29	0.014
Pancreas texture (soft)	0.91	2.47	1.26–4.85	0.008

### Predictive nomogram for POPF

**Fig. 1:**
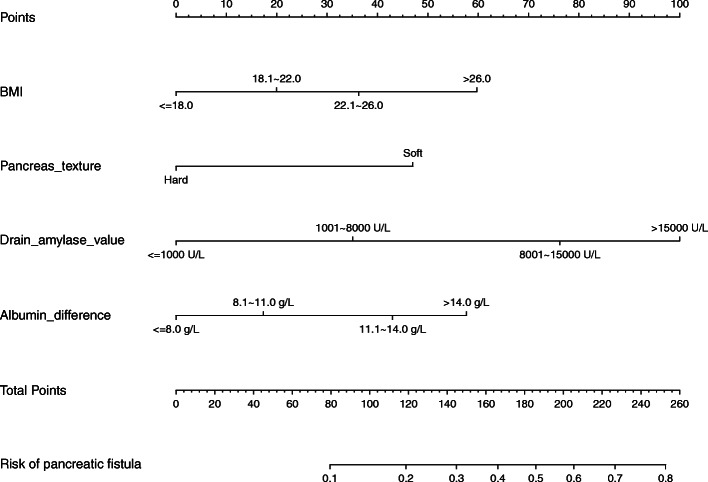
The predictive nomogram of postoperative pancreatic fistula

A nomogram was established based on the results of the logistic regression analysis (Fig. 1). The points of each predictor in the nomogram were obtained from the point axis. The estimated probability of POPF was subsequently determined by the sum of all the points from all predictors.

### Internal validation of the nomogram

**Fig. 2:**
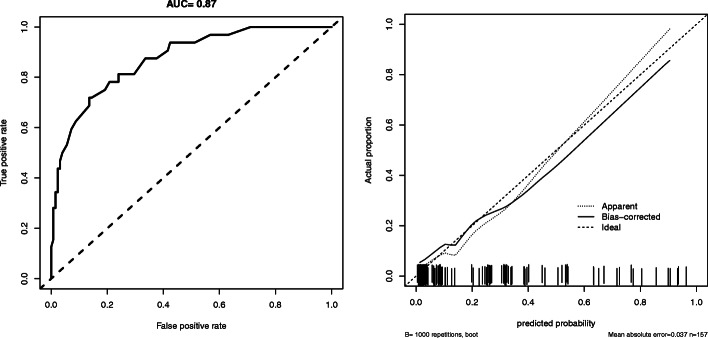
The ROC curve and calibration curve of the nomogram

The performance of the nomogram was further validated in the validation cohort (Fig. 2). In our validation dataset, the C-index was 0.87, indicating good discrimination ability (Fig. 2a). The calibration curves for the probability showed good concordance between the predicted and actual observations (Fig. 2b). These results indicate that the nomogram shows accurate predictive power.

Figure 2a The AUC under the ROC curve of the model was approximately 0.87 and showed good discrimination. Figure 2b The calibration curve of the nomogram showed accurate predictive ability

### Comparison of the nomogram and the alternative fistula risk score

The predictive value of the nomogram, including AUC, accuracy, sensitivity, specificity, negative predictive value, and positive predictive value, was compared with that of the widely used a-FRS model [13]. The nomogram revealed a better predictive ability than the a-FRS model (AUC = 0.87 vs 0.62, *P* < 0.001). Moreover, the other parameters (sensitivity, specificity, accuracy, negative predictive value, positive predictive value) of the nomogram were better than those of the a-FRS model (details are listed in Table 3).

**Table 3:** Predictive value of the nomogram and the a-FRS

Models	AUC	95% CI	P*	Sensitivity	Specificity	PPV	NPV	Accuracy
Nomogram	0.87	0.81–0.94	< 0.001	81.25%	76.00%	46.43%	94.06%	77.07%
a-FRS	0.62	0.52–0.73		65.63%	51.20%	25.61%	85.33%	54.14%

*PPV* negative predictive value, *NPV* positive predictive value, *a-FRS* alternative fistula risk score. *the AUC of nomogram vs the AUC of a-FRS

## Discussion

According to the latest definition of pancreatic fistula, patients can experience POPF as early as POD 3. Given that the predictive power of the models that include only preoperative variables, we aimed to establish a predictive model of POPF that can help physicians to effectively screen high-risk patients on POD 1. In this study, we developed a nomogram that includes AD, pancreatic texture, DAV, and BMI, and the nomogram demonstrated good predictive ability. Except for pancreas texture, the other three predictors in the model are objective. The predictors included in this model are accessible and universal.

Preoperative hypoalbuminemia is believed to be a risk factor for POPF following PD [23]. In our work, the level of preoperative serum albumin was in the normal range, and no significant difference was found between the two groups. However, we identified AD as a strong independent predictor of POPF. AD has been proven to be a risk factor of POPF after pancreatectomy [17, 18]. To some extent, AD can reflect the overall postoperative changes in patients. As a negative acute-phase protein, albumin is decreased during the acute disease phase. Previous studies have demonstrated that the AD is up to 10–15 g/L [16], and our result is consistent with this. Leakage into the third space and intercellular space and increased catabolism may be the main causes of a high AD [16, 17]. Albumin performs many physiological functions, including anti-inflammatory activities, providing nutrition and energy to fast-growing tissues, and stimulating repair or remodeling [17]. Moreover, AD is closely associated with cardiac loading and tissue edema, which are also not conducive to tissue repair [24].

The DAV on POD 1 was another important predictor of POPF. In accordance with our results, a study of 463 patients who underwent PD showed that a DAV on POD 1 > 500 U/L was associated with a 21.72-fold increase in pancreatic fistula risk [25]. Several other studies also emphasized the DAV on POD 1 in predicting POPF [6, 8, 26]. There was also a report that most of the patients with a higher DAV after the initial postoperative days could experience an uneventful course even if the value decreased to a normal level [26]. A higher DAV on POD1 may be representative of exuberant secretion function and pancreatic juice leakage volume.

The soft pancreatic parenchyma is an accepted factor for POPF after PD and has been included in many predictive models [13, 23]. A prospective validation study showed that a soft pancreas is believed to be more prone to developing POPF (*P* = 0.002) [7]. The texture of the pancreas is positively associated with contained acini [27]. Fibrotic pancreatic tissue, usually with acini regeneration and impaired secretion function, is believed to be less prone to pancreatic leakage [28]. Moreover, a soft pancreas is much more likely to be injured during surgery. However, the pancreas texture is an objective factor, and no uniform standard for assessment has been reached.

BMI as a risk factor has also been accepted in a number of articles [29]. BMI can reflect the amount of body fat, and a higher BMI usually means that a person carries more fat. Visceral fat has also been proven to be a predictor of POPF. Large amounts of proinflammatory cytokines are contained in fat tissue. When fat tissue is damaged, the released cytokines are not conducive to tissue repair. Moreover, a higher BMI is positively related to intraoperative technical difficulties and pancreatic fatty infiltration [30].

More than ten predictive models of POPF after PD have been established [10, 29]. However, the performances of these models are questionable [13]. The widely used a-FRS model also showed a limited predictive capability in our study, which is in accordance with previous studies [9, 10]. Nomograms have been used in several cancers and are very simple to use, the risk of POPF can be assessed directly on the nomogram without the help of a calculator. In this study, AD was identified as a new, accessible predictor, and it was strongly associated with the occurrence of POPF. The majority of reported predictors, including pre-, intra- and postoperative variables, were considered in this analysis, which makes it more convincing. Thus far, all the predictors are variables that can only reflect the physiological state at a certain moment. AD is a variable that capable of reflecting dynamic changes in patients, and it may be a representation of the body’s ability to adapt to stress.

The predictors in this model are universal and accessible. We believe that this model might enable surgeons to make early and accurate prediction of POPF and to make a clinical management decision. First, albumin is widely used clinically; exogenous albumin infusion might benefit those with a higher AD after PD. Second, DAV on POD1 was identified as a risk factor of POPF, and somatostatin and its analogues may be a reasonable choice for treating selected patients. Third, for patients with soft pancreases, a pancreatic duct stent should be placed and anastomotic firmness should be enhanced. In addition to helping surgeons reduce the incidence of pancreatic fistula, this model can help physicians to develop strategies for accelerating recovery in low-risk populations, such as early drain removal for patients with a low risk of POPF. We believe that as long as clinicians utilize this model reasonably, the incidence of POPF can be decreased.

Our study has its own limitations. First, as a retrospective study performed at a single institution, this study was subject to selection bias and confounders. However, to to overcome the selection bias, consecutive patients were included, and specific exclusion criteria and regression model were used to remove the confounding factors. Second, some data that were not available in this study may be important factors for POPF, such as blood loss. Third, this model was not externally validated. Moreover, due to the eliminate sample size; we did not perform a stratified analysis of the surgical procedure. The limitations weaken the credibility of this study. In the future, a multicenter prospective study should be carried out, and the predictive model should be externally validated.

## Conclusions

We have established and internally validated a nomogram that includes AD, pancreatic texture, DAV, and BMI. The nomogram showed a good performance and could help doctors to screen high-risk patients on POD 1. AD was identified as a new and accessible predictor of POPF following PD.

## Data Availability

All data analysed during this study are included in this published article.

## Abbreviations

AD: Albumin difference
a-FRS: Alternative fistula risk score
AUC: Area under curve
PD: Pancreaticoduodenectomy
POD: Postoperative day
POPF: Postoperative pancreatic fistula
ROC: Receiver operating characteristic curve
